# Genetic CJD with a novel E200G mutation in the prion protein gene and comparison with E200K mutation cases

**DOI:** 10.1186/2051-5960-1-80

**Published:** 2013-12-12

**Authors:** Mee-Ohk Kim, Ignazio Cali, Abby Oehler, Jamie C Fong, Katherine Wong, Tricia See, Jonathan S Katz, Pierluigi Gambetti, Brianne M Bettcher, Stephen J DeArmond, Michael D Geschwind

**Affiliations:** 1 Department of Neurology, Memory and Aging Center, University of California, San Francisco (UCSF), San Francisco, CA 94143, USA; 2 National Prion Disease Pathology Surveillance Center (NPDPSC), Case Western Reserve University, School of Medicine, Cleveland, OH 44106, USA; 3 Department of Clinical and Experimental Medicine, Second University of Naples, Naples, Italy; 4 Department of Pathology, UCSF, San Francisco, CA 94117, USA; 5 Forbes Norris ALS/MDA Center, Department of Neurology, California Pacific Medical Center, 2324 Sacramento St, Suite 111, San Francisco, CA 94115, USA

**Keywords:** Creutzfeldt-Jakob disease, E200K, familial CJD, Synaptic PrP^Sc^, Curvilinear PrP^Sc^

## Abstract

A novel point mutation resulting in a glutamate-to-glycine substitution in *PRNP* at codon 200, E200G with codon 129 MV polymorphism (cis valine) and type 2 PrP^Sc^ was identified in a patient with a prolonged disease course leading to pathology-proven Jakob-Creutzfeldt disease. Despite the same codon as the most common genetic form of human *PRNP* mutation, E200K, this novel mutation (E200G) presented with a different clinical and pathological phenotype, including prolonged duration, large vacuoles, no vacuolation in the hippocampus, severe neuronal loss in the thalamus, mild cerebellar involvement, and abundant punctate linear and curvilinear deposition of PrP^Sc^ in synaptic boutons and axonal terminals along the dendrites.

## Background

Human prion diseases are unique in medicine as they occur as spontaneous, genetic and acquired forms. Sporadic Jakob-Creutzfeldt disease (sCJD) is the most common human prion disease, accounting for approximately 85-90% of cases, whereas autosomal dominant genetic forms, due to more than 30 mutations in the prion protein gene (*PRNP*), account for 10-15% of cases [1]. Glu200Lys (E200K) and Asp178Asn (D178N) are the most common *PRNP* mutations worldwide [2-8]. At least four founder groups of E200K are known with the two largest populations in the Middle East (Libyan Jews) and Slovakia [4,5,8-10]. We report a novel point mutation of *PRNP* at codon 200 resulting in a glutamate-to-glycine substitution, E200G, in a pathology-proven patient of British descent and compare findings to those of E200K cases.

## Case presentation

A 59-year-old Caucasian woman with a reported 25-month history of motor and cognitive problems was referred to our clinical research center with suspected sCJD. Her first obvious symptoms began 25 months prior (“onset”) with gradual onset of gait imbalance, fatigue and “loss of mental acuity,” although there might have been very subtle changes in personality (irritability, decreased understanding and appreciation of humor, poor planning) even five months earlier. One month after onset, she developed difficulty walking. By four months, she could no longer correctly balance her checkbook and had worsened handwriting. At six months, language difficulties began, particularly noticeable during phone conversations. At seven months, she began missing freeway exits (reasons unclear). At 12 months, she developed a head tremor and at 17 months, nighttime leg cramps. By 18 months, all of the above symptoms had worsened, and she required a cane. At 20 months, she still was doing some, albeit more limited, driving, shopping, and light housework, with some fluctuation of her gait and memory abilities. By 22 months, she could no longer do these activities. At 25 months, when she first visited our center, she was wheelchair bound and had difficulty following conversations.

**Figure 1:**
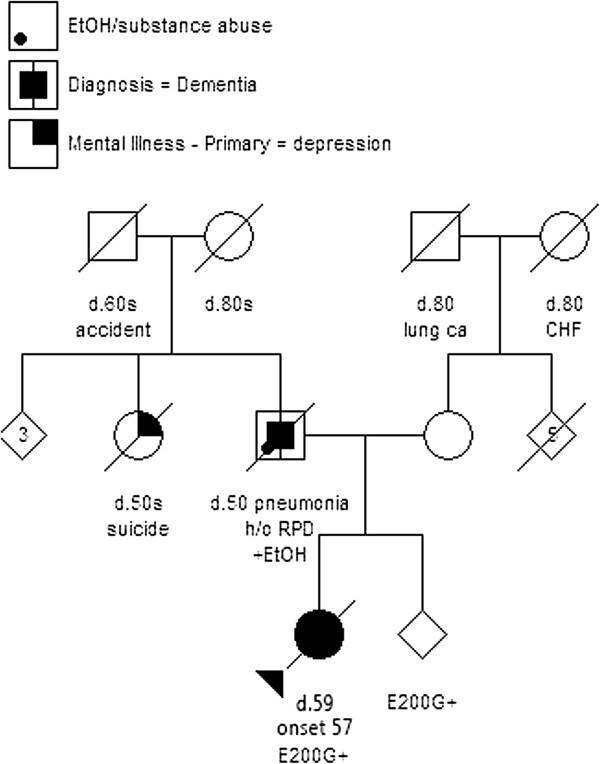
**Family pedigree.** Family pedigree of the proband of British descent with E200G mutation. Circles indicate females, and squares indicate males. Those whose gender is not disclosed are indicated with rhombi. A diagonal bar in the symbols indicate deceased. Arabic numerals “3” and “5” indicate the numbers of individuals. The proband with the E200G mutation (E200G+) is indicated by closed circle and arrowhead and her sibling with the same E200 mutation is also marked by “E200G+” (+EtOH = alcoholism, d. = died at age, lung ca = lung cancer, CHF = congestive heart failure, h/o RPD = history of rapidly progressive dementia).

Her past medical history was unremarkable. Her father died at 50 from alcoholism and pneumonia, with presumed “alcohol-related dementia,” which progressed rapidly during the last three months and “fast movements” during his last 6 weeks. The proband had an unaffected sibling, 11 years younger, and a half-sibling whose status is unknown (Figure 1).

At 25 months, she was alert and partially oriented, fluent, slightly hypophonic, but not dysarthric. She had mild anomia. Her MMSE was 26/30, missing points for floor, county and recall. More extensive neuropsychological testing revealed mild cognitive impairment across multiple domains, including visual memory and executive function (Table 1). Examination also was remarkable for jerky ocular pursuit (horizontal and vertical), increased latency with mild decreased velocity of horizontal saccades, extrapyramidal features (resting tremor in the face and bilateral upper extremities, action tremor in the bilateral upper extremities, mild cogwheel rigidity in the bilateral upper extremities, and bradykinesia greatest in the right arm and left leg), symmetric, distal, length-dependent, decreased pinprick and temperature in legs, asymmetric lower extremity reflexes (left side brisker), and cerebellar dysfunction (wide-based gait requiring assistance and truncal ataxia). There was no limb ataxia, myoclonus, dystonia, chorea, alien limb, or higher cortical sensory signs.

**Table 1:** Serial longitudinal neuropsychological assessment of E200G case

	**Visit 1**	**Visit 2**	**Visit 3**
	**25 months**	**26 months**	**28 months**
	**Raw (Z-score)**	**Raw (Z-score)**	**Raw (Z-score)**
**Global cognition**			
MMSE	26^1^	24^2^	16
**Memory**			
CVLT trial 5	4 (−4.00)	4 (−4.00)	0 (−5.00)
CVLT delay	1 (−4.00)	1 (−4.00)	0 (−4.00)
CVLT recog hits	15 (0.00)	15 (0.00)	13 (−1.00)
CVLT recog false pos	3 (−0.50)	3 (−0.50)	16 (−4.00)
Benson figure delay recall	3 (−4.17)	0 (−5.48)	N/D
*Mean memory Z-score*	−2.53	−2.80	−3.50
**Executive function**			
Modified trails	59" (−3.00)	119" (−8.62)	120" (−8.71)
Design fluency	N/A	5 (−2.28)	0 (−4.07)
Digit backwards span	3 (−1.85)	3 (−1.85)	3 (−1.85)
Stroop inhibition	31 (−2.25)	19 (−3.50)	N/D
*Mean executive Z-score*	−2.37	−4.06	−4.88
**Language**			
BNT-Abbrev	13 (−1.66)	15 (+0.55)	N/D
**Visuospatial**			
Benson figure copy	13 (−1.27)	14 (−1.36)	N/D

^1^ Missed 2 points for orientation and 2 on recall. ^2^ Missed 3 points for orientation and 3 on recall. *Abbreviations and Definitions:MMSE* Mini-Mental State Examination, *CVLT* California Verbal Learning Test-II (16 word list), *Recog* Recognition, *False Pos* False Positive Total, *Modified Trails* Abbreviated trail-making task using numbers and days of the week, *BNT-Abbrev* Abbreviated (15-item) Boston Naming Test, *N/D* patient unable to perform, *N/A* not administered.

**Figure 2:**
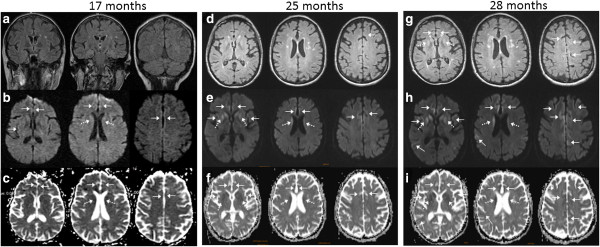
**Serial brain MRIs, classic for CJD.** Brain MRIs at 17 **(a-c)**, 25 **(d-f)**, and 28 **(g-i)** months. Solid arrows indicate cortical ribboning. Dotted arrows indicate deep nuclei hyperintensity. **(a)** Initial brain MRI was read by us as “ambiguous” for CJD, but was felt to show mild frontal-parietal atrophy and punctate subcortical white matter hyperintensities on FLAIR **(b)** DWI shows subtle cortical ribboning in the anterior and median cingulate and right posterior cingulate and subtle striatal hyperintensity. **(c)** ADC map shows hypointensities in the same regions as on DWI. **(d)** FLAIR (axial) brain MRI at 25 months reveals moderate diffuse cortical atrophy. **(e)** DWI now shows cortical ribboning in the right frontal cortex, and anterior and median cingulate, and hyperintensities in bilateral caudate and putamen with anterior to posterior gradient. **(f)** ADC map shows hypointensities in the areas of DWI hyperintensity. **(g)** FLAIR MRI at 28 months shows more prominent cingulate cortical ribboning hyperintensity in the striatum. **(h)** Cortical ribboning is more prominent than three months prior. **(i)** ADC map shows hypointensities consistent with restricted diffusion.

Her initial brain MRI at 17 months showed subtle cortical ribboning and striatal hyperintensity on FLAIR and DWI with restricted diffusion on ADC, which were more distinct and extensive by 25 months (Figure 2a-f). Her first EEG, at 25 months, showed bilateral slowing, predominant in the frontal and posterior regions, but no triphasic or epileptiform discharges. Extensive blood tests to rule out conditions other than CJD were negative. Basic CSF findings at 25 months were normal (RBC 1, WBC 1, protein 37, glucose 75, no oligoclonal bands, and IgG index of 0.5), except she had an elevated total tau of 1351 pg/mL (“>1150 pg/ml consistent with CJD,” NPDPSC), ambiguous 14-3-3 protein (NPDPSC) and mildly elevated (“intermediate”) neuron-specific enolase of 27 ng/mL (Mayo Laboratories; normal < 15, intermediate 15–35, >35 ng/ml consistent with CJD). Follow-up neuropsychological testing one and three months later (at 26 and 28 months) showed further deterioration in memory and executive function (Table 1). Her brain MRI at 28 months showed profound cortical atrophy, brighter and more extensive DWI hyperintensities with increased restricted diffusion in these regions (Figure 2g-i). She passed away at 30 months, 5 months after diagnosis. Direct sequencing of the *PRNP* gene open reading frame, and subsequent cloning experiments (NPDPSC), from blood (and later from frozen brain tissue) revealed an E200G mutation with heterozygosity at codon 129 (MV; cis valine) in *PRNP*. Her younger asymptomatic full sibling carried the same mutation and codon 129 polymorphism.

**Figure 3:**
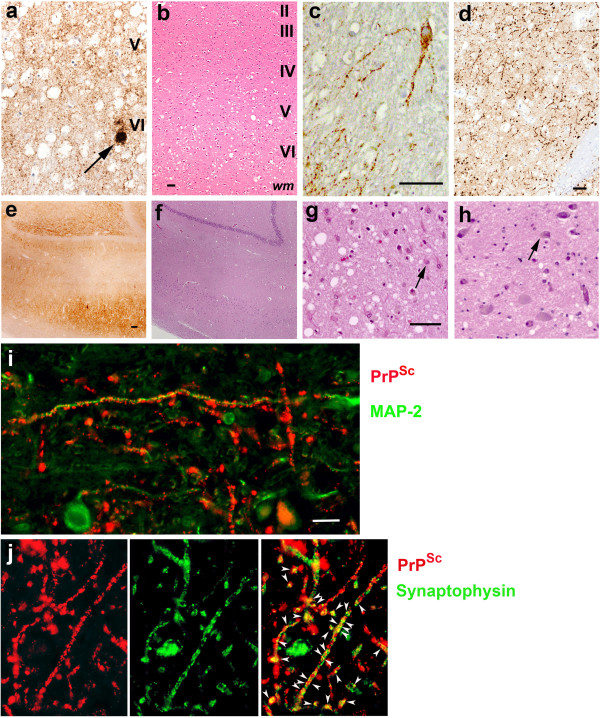
**Neuropathological immunohistochemistry of the E200G and a representative E200K case for comparison. (a-g E200G; h E200K) (a-b)** insular cortex, **(c)** dentate nucleus, **(d, i-j)** putamen, **(e, f)** hippocampus, **(g)** thalamus, **(h)** E200K-129M thalamus. **(a)** Finely granular (synaptic) pattern of PrP^Sc^ accumulation occurs in layers V-VI, with a few larger PrP^Sc^ deposits (arrow). **(b)** H & E stain reveals vacuolation mostly in cortical layers V-VI. **(c)** A cerebellar dentate nucleus neuron shows granular deposits of PrP^Sc^ inside and around the perikarya and processes. **(d)** Many punctate linear and curvilinear arrays of PrP^Sc^ were located in the putamen. **(e)** Moderate to severe PrP^Sc^ accumulation is shown in the hippocampus, where **(f)** H & E stain shows no vacuolation (the same area as E). **(g)** H & E stain shows severe neuronal loss and reactive astrocytosis with mild vacuolation in the medial nucleus of thalamus (a representative astrocyte marked with an arrow). **(h)** H & E stain shows small vacuoles but neither neuronal loss nor reactive astrocytosis in the medial nucleus of thalamus of an E200K-129M patient (a representative neuron marked with an arrow). **(i)** PrP^Sc^ (red fluorescence) in the linear and curvilinear arrays contacts dendrites stained for MAP-2 (green fluorescence) along its course. **(j)** Some of PrP^Sc^ (red fluorescence) in the arrays is co-localized (yellow fluorescence, arrowheads) with synaptophysin (green fluorescence). Other PrP^Sc^ staining, however, is not co-localized, but in close proximity to synaptophysin., suggesting it is near pre-synaptic axonal terminals. Bars below **b** and **c**, 50 μm. Bar below **d**, 30 μm applying also to **a**. Bar below **e**, 100 μm, applying also to **f**. Bar below **g**, 50 μm applying also to **h**. Bar below **i**, 20 μm applying also to **j**. Layers of the cerebral cortex are indicated. “wm” indicates white matter.

**Figure 4:**
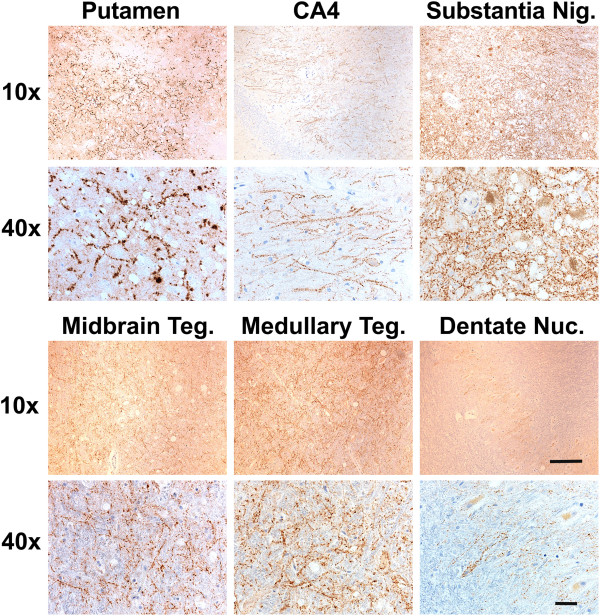
**Punctate linear and curvilinear arrays of PrP**^**Sc **^**in various brain regions.** The PrP^Sc^ stain shows the punctate linear and curvilinear deposits of PrP^Sc^ in the putamen, hippocampus CA4, substantia nigra (Substantia Nig.), midbrain tegmentum (Midbrain Teg.) medullary tegmentum (Medullary Teg.), and cerebellar dentate nucleus (Dentate Nuc.) at low (10x) and high (40x) magnifications. Bar below 10x is 150 μm and bar below 40x is 50 μm.

Autopsy was performed one day after her passing. The brain weighed 1175 gm (normal 1100–1400 gm). Gross brain pathology showed atrophy in the frontal lobe, right greater than left, and loss of pigmented neurons in the substantia nigra. Microscopic examination revealed PrP^Sc^ deposition characteristic of CJD, with both perivacuolar and diffuse finely granular synaptic staining in the cerebral cortex (Figure 3a). Most PrP^Sc^ consisted of finely granular deposits (2–4 μm diameter), which were distributed around and, occasionally, inside the neuronal perikarya and processes (Figure 3c). Occasional larger plaque-like deposits were also seen (30–40 μm) (Figure 3a). In addition there were abundant punctate, linear and curvilinear arrays of PrP^Sc^ in the striatum (Figures 3d and 4) and other regions including Ammon’s horn of the hippocampus, substantia nigra, midbrain tegmentum, periaqueductal gray, and medullary inferior olivary nucleus (Figure 4). MAP-2 immunostaining showed the linear and curvilinear arrays were associated with dendrites (Figure 3i). PrP^Sc^ was not co-localized with axonal markers including neurofilament H and phospho-neurofilament H (not shown). Some of the unusual linear and curvilinear PrP^Sc^ deposits co-localized with or were adjacent to synaptophysin (presynaptic vesicle membrane protein) immunostaining, demonstrating the presence of PrP^Sc^ in synaptic boutons and probably also in pre-synaptic axonal terminals (Figure 3j).

In most regions of the cortex, vacuolation was in layers II-VI, more severely affecting layers III-VI (not shown), but in the insular cortex, it was confined to layers V and VI (Figure 3b). The mean diameter of gray matter vacuoles was 14.9 μm (range 4–108 μm), significantly larger than those observed in the NPDPSC E200K-129V (mean: 11.9 μm, range: 4–72 μm; P < 0.016) and E200K-129M (mean: 11.8 μm, range: 4–69 μm; P < 0.016) cohorts. Vacuole size between the E200K-129V and -129M NPDPSC cohorts was not significantly different (P > 0.016). Reactive astrocytosis co-localized with vacuolation and PrP^Sc^ deposition in all cortical regions and striatum, except in the insular cortex, where it was most intense in layers I-IV that contained sparse vacuolation and minimal PrP^Sc^ (not shown).

The hippocampus did not exhibit vacuolation, or neuronal loss despite mild to severe PrP^Sc^ deposits; furthermore astrocytosis was absent or moderately reactive (Figure 3e, f, Table 2). The thalamus showed overall mild vacuolation and mild to severe PrP^Sc^ deposits, severe neuronal loss, and severe reactive astrocytosis, especially in the medial nuclei (Figure 3g, Table 2); in contrast, the thalami of the NPDPSC E200K-129M and -129V cases showed vacuoles but neither neuronal loss nor astrocytosis (Figure 3h). All brain regions were affected to a moderate or severe extent, except the cerebellum, which only had sparse PrP^Sc^ staining, no vacuolation, and mild reactive astrocytosis in the cortex (not shown). In the cerebellar molecular layer, there were loose, round aggregates of fine granules, which occasionally were around cell bodies (not shown). Plaque-like PrP^Sc^ deposits were observed in the granular cell layer, whereas perineuronal and intraneuronal staining patterns were predominant in the dentate nucleus, which also showed a moderate number of linear and curvilinear arrays (Figures 3c and 4). Table 2 shows lesion profiles of the E200G case.

**Table 2:** Lesion profiles of the E200G case

**Pathology\ Brain regions**	**PrP**^**Sc **^**deposition**^**a**^	**Vacuolation**^**b**^	**Reactive astrocytosis**^**c**^
Frontal	Layers 5, 6 ++/+++ (S/PL/FG)	Layer 6 +++	Pattern1: layer 1 +
		Layers 2–5 ++	Pattern 2: layers 1–4 ++
	Layers 1–4 + (FG)		Pattern 3: layers 1–6 +++
Parietal	Layers 5, 6 ++	Layers 5–6 ++	Pattern1: layer 6 ++
	(S/PL/FG)	Layers 2–4 +	Pattern 2: layers 2–6 ++
	Layers 1–4 + (FG)		
Temporal	Layers 5, 6 ++/+++ (FG)	Lattern 1:	Pattern 1: layers 1–5 ++++
	Layers 1–4 ++ (FG)	Layers 3–6 +++	Pattern 2: layers 1–5 +/++
		Layer 2 SS	
		Pattern 2:	
		Layers 5–6 +++	
		Layers 2–4 ++++ (SS)	
Occipital	Layers 5, 6 + (FG)	Layer 6 +	Layers 1–6 −/+
	Layers 1–4 −/+ (FG)		
Cingulate	Layers 1–6 +++ (FG)	Layers 2–3 ++	Layers 2–6 ++++
		Layers 4–5 +++	
		Layer 6 ++	
Insula	Layers 5, 6 +++ (FG)	Layers 5–6 +++	Layers 1–4 +++
	Layers 1–4 ++ (FG)	Layers 2–3 ++	Layers 5, 6 −
Caudate	+++ (S, FG)	+++	+++
Putamen	+++ (S, FG)	+++	+++
Globus pallidus	Ext. +++ (FG)	Ext. +	Ext.++/+++
	Int. ++/+++ (FG)	Int. +	Int. ++
Thalamus^d^	Rostral: med.+/++ (S), lat. −	Rostral: med.++, lat.−/+	Rostral: med.++/+++, lat. +/++
	Middle: med. + (S), lat. −	Middle: med.++, lat.+	Middle & caudal: med. ++/+++, lat. −/+
	Caudal: med. 1+ but focal 2+, lat. -	Caudal: med.++/+++, lat. +	
Amygdala	++/+++ (FG)	+++	++/+++
Hippocampus^d^	Dentate gyrus, CA4, CA3, and CA2: −/+	CA4, CA3, CA2, and CA1 prox. –	CA4, CA3 ++
			CA2, CA1 prox. –
	CA1: prox. ++, CA1 dist.+++ (FG)	CA1 dis. ++	CA1 dis. ++,
	Subiculucm: +++	Subiculum:	Subiculum ++,
	Entorhinal cortex: layers 5, 6 ++/+++	Layer 4 +++	Entorhinal cortex: layers 2–6 ++
		Layer 2 +/++	
	Layers 1–4 −/++ (FG)	Entorhinal cortex	Transentorhinal cortex: layers 2–6 +++
	Transentorhinal cortex: layers 1–6 +++ (FG)	: layers 2,4 +++	
		Transentorhinal cortex	
		: layers 2–6 +++	
Cerebellum	Molecular and granular layers −/+	Molecular and granular Layers – dentate nucleus +	Molecular layer ++/+++
			Granular layer +/++
			Dentate nucleus +
	Dentate nucleus +		
Midbrain	Periaqueductal gray and tegmentum + (FG)	Periaqueductal gray ++	Periaqueductal gray +
		Tegmentum +	Tegmentum ++/+++
Substantia nigra	++/+++ (FG)	++	+/++
Medulla	Tegmentum ++ (FG)	Tegmentum +	Tegmentum +
	Inf. olive ++/+++ (FG)	Inf. olive -	Inf. olive +

^a^ PrP^Sc^ deposition scored on a 4-level scale: - absent, + mild, ++ moderate, +++ severe; ^b^ Vacuolation scored on a 5-level scale - absent, + mild, ++ moderate, +++ moderately severe, ++++/SS very severe/status spongiosis. ^c^ Reactive Astrocytosis scored on a 5 -point scale - absent, + mild, ++ moderate, +++ moderately severe, ++++ very severe. ^d^ data shown is from UCSF pathological analysis. NPDPSC analyses were generally in agreement with UCSF analyses except NPDPSC found +++ PrP^Sc^ in medial thalamus and hippocampus. *Abbreviations:S* Synaptic, *PL* Plaque-like, *FG *Finely granular, *SS* Status spongiosus, *ext.* Externa, *int.* Interna, *med.* Medial, *lat.* Lateral, *prox.* Proximal, *dis.* Distal, *inf.* Inferior.

**Figure 5:**
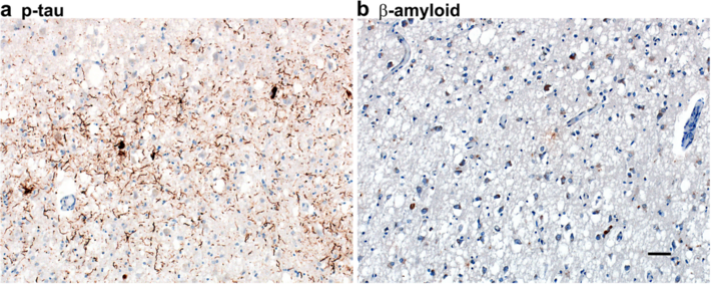
**Hyperphosphorylated tau and β-amyloid (Aβ) immunoreactivity in the E200G case. (a)** Many hyperphosphorylated tau positive neuropil threads and four neuronal cell bodies are found in the entorhinal cortex layers 3 and 4. **(b)** No Aβ plaques are seen but many neuronal cell bodies are Aβ positive in the entorhinal cortex layers 3 and 4. Bar in **b** represents 100 μm and applies also to **a**.

Because other neurodegenerative proteins occasionally are found in sCJD and gPrD [11-15], we examined the status of hyperphosphorylated tau (Hτ), β-amyloid (Aβ), α-synuclein, TPD 43, and ubiquitin in our patient. The CP13 antibody specific for Hτ was used to stain sections from the insula and putamen, the hippocampus and entorhinal cortex (EC), the inferior temporal lobe, and the midbrain with substantia nigra. Hτ was found exclusively and focally in layers 3 and 4 of the EC (Figure 5a). Application of the 4G8 antibody for abnormal Aβ neuritic and amyloid plaques in Alzheimer’s disease (AD) did not reveal Aβ plaques in our patient; many neuronal cell bodies, however, were Aβ immunopositive in the entorhinal cortex layers 3 and 4 (Figure 5b). With the α-synuclein antibody, no abnormal α-synuclein in the form of classical Lewy bodies was found in the substantia nigra, as verified by the H&E stain, or as cortical Lewy bodies (not shown). No displacement of TDP43 from the nucleus to the cytoplasm or neurites was identified in any brain regions examined (not shown). Ubiquitin and PrP^Sc^ double immunofluorescence staining of the curvilinear PrP^Sc^ deposits in the putamen and the cerebellar dentate nucleus did not show ubiquitin positive structures (not shown).

**Figure 6:**
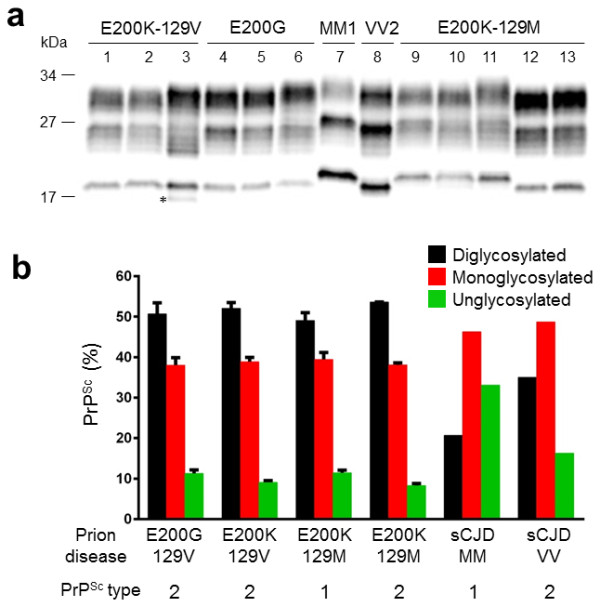
**Prion typing.** Determination of electrophoretic mobility and PrP^Sc^ glycoform ratio in gPrD with E200G and E200K mutations. **(a)** Western blot analysis of PrP^Sc^ from an E200K-129V patient (lanes 1–3), our E200G case (lanes 4–6), an E200K-129M patient associated with PrP^Sc^ type 1 (lanes 9–11) and an E200K-129M patient associated with PrP^Sc^ type 2 (lanes 12–13). The mobility of the unglycosylated PrP^Sc^ from E200G is ~ 19 kDa as that of PrP^Sc^ type 2 and matches the corresponding mobility observed in E200K-129V and E200K-129M associated with PrP^Sc^ type 2. A fragment of ~17 kDa (indicated by the asterisk) was detected in the cerebellum of the two E200K-129V. PrP^Sc^ from sCJDMM1 (lane 7) and sCJDVV2 (lane 8) subtypes have been loaded as control. Frontal (lanes 1, 4, 7–9 and 12), parietal (lane 5), occipital (lanes 2, 10 and 13) cortices and cerebellum (lanes 3, 6 and 11) were examined. **(b)**The amounts of di-, mono- and unglycosylated PrP^Sc^ glycoforms expressed as percentage of the total PrP^Sc^ are virtually identical in all cases associated with the E200G and E200K mutations, but they differ from those of sCJDMM1 and sCJDVV2. Bar graphs are expressed as mean ± SEM of the two cortical regions from cases of E200K-129M (n = 3), E200K-129V (n = 4), and E200G, and one region from sCJDMM1 (n = 1) and sCJDVV2 (n = 1).

Western blot analysis of the proteinase K (PK)-resistant PrP (PrP^Sc^) from our patient showed PrP^Sc^ with gel mobility of the unglycosylated PrP^Sc^ isoform of ~19 kDa, matching PrP^Sc^ type 2 (Figure 6a). There was prominent representation of diglycosylated PrP^Sc^ and underrepresentation of unglycosylated PrP^Sc^ species (Figure 6a).

### Comparison to E200K cases evaluated through the NPDPSC

To compare the clinical and pathological phenotype of our E200G-129V(M) (trans M) case to that of E200K, we selected three E200K-129V(M) cases for whom sufficient clinical data were available from among the six NPDPSC cases used for histopathological examination. Table 3 summarizes the clinical and pathological findings of these NPDPSC E200K-129V(M) cases, ages 37–67. These three NPDPSC E200K-129V(M) cases all initially manifested with gait difficulty followed by memory decline, and progressed rapidly over 7–9 months. One case had myoclonus in the arms and two cases showed slowing on EEG. All three cases reportedly showed reduced striatal diffusion on brain MRI but no definitive cortical ribboning was reported (although cortical ribboning is often missed [16]). Pathological examination of these three NPDPSC E200K-129V(M) cases showed classical features of CJD throughout the cerebral cortex, striatum, hippocampus, and cerebellum and type 2 PrP^Sc^. These three cases (as well as the three other histopathologically-analyzed cases without sufficient clinical history) also showed mild to moderate, punctate linear and curvilinear PrP^Sc^ deposits in the midbrain and cerebellar dentate nuclei. In addition, this punctate linear and curvilinear PrP^Sc^ deposition pattern was also observed in most E200K-129M(V) cases (8 of 9 cases examined), particularly in the putamen, globus pallidus, midbrain, cerebellar dentate nucleus, and hippocampus (Table 4).

**Table 3:** **Clinical features of NPDPSC E200K**-129V (trans M) cases*

	**Patient C**	**Patient E**	**Patient F**
Age/gender	37 yo/male	57 yo/female	67 yo/female
Age of onset	37 yo	57 yo	67 yo
Duration of illness	9 months	8 months	7 months
First symptom	Gait difficulty	Gait difficulty and nonspecific dizzy feeling	Veering to the left and worsening gait
Other symptoms and cognitive tests	Speech difficulty; dysarthria	Blurry and double vision at 2–3 months	Weak voice,
			Hands tremor,
	MOCA 19/30 at 4 months	Curled hands, clumsy hands	Unable to write,
			Short term memory decline at 2–3 months
		Cognition deterioration	MMSE 25/30 (no recall, no copy of design) at 4 months,
		MMSE 19/30 (missing points on orientation and recall) at 4 months	
			MOCA 10/30 at 4 months
			MMSE 27/30 at 5 months (remote recall 0/3)
Myoclonus and other abnormal neurological findings	Myoclonus + in arms	Myoclonus - vertical gaze palsy+	Myoclonus - postural tremor+, bradykinesia +
EEG	N/A	Intermixed bursts of delta and theta activity at 4 months	Right temporal slowing at 4 months
Brain MRI	Read normal at 2 months.	Reported as only periventricular white matter changes at 2 month.	Reported as high T2 signal in the cerebellar gray matter and vermis, cerebellar atrophy at 3 months.
	At 3 months, reported as **symmetrical T2 and DWI hyperintensity in the caudate, putamen, and pulvinar**	**Restricted diffusion in the caudate at 4 months**	At 4 months, **restricted diffusion in the caudate bilaterally, subtle restriction in the thalamic pulvinar area**, left greater than right, and questionable high cortical intensity in the mesial frontal and parietal high convexities on DWI but no corresponding ADC findings, increased signal in the entire cerebellum on DWI
Other imaging	Unremarkable C, T, and L spine MRIs	Disc bulging on C-spine MRI	Normal pelvic US and breast MRI
CSF profile	Clear, colorless, WBC 0, protein 61(15–60),	**Positive 14-3-3**	Clear, colorless, WBC 0, protein 46 (12–60), glucose 84 (40–70)
		**tau 14746 pg/mL**	**14-3-3 4.5 ng/mL**
	glucose 65 (45–75)		(nl < 1.5 ng/mL, Mayo clinic)
	**Positive 14-3-3**		CSF paraneoplastic panel: negative (Mayo clinic)
	**tau 20360 pg/mL**		
Other labs	Serum paraneoplastic panel negative		Serum paraneoplastic panel negative
PrP^Sc^ type	2	2	2
Family history for CJD	Unknown	Positive for progressive movement problems and dementia: 2 family members + for genetic testing for prion disease	Father died of CJD at age 69 within 6 months
Histological and immunohistochemical findings	**1.Vacuolation and reactive astrocytosis**	**Similar to patient C but** with less intense PrP^Sc^ immunostaining and no plaque-like PrP ^Sc^ formations.; Global cerebellar atrophy	**Similar to patient C but** with less intense PrP ^Sc^ immunostaining and no plaque-like PrP ^Sc^ formations.; Focal cerebellar atrophy
	1) Intense in the cerebral neocortex, basal ganglia, hippocampus, and cerebellum		
	2) Mild to moderate vacuolation in the thalamus		
	3) Vacuolation more severe in the basal ganglia than the cerebral cortex and thalamus		
	**2. PrP**^**Sc **^**deposits**		
	1) Diffuse and plaque like deposits in the cerebral cortex		
	2) Presence of intraneuronal PrP^Sc^ deposit		
	3) Moderate PrP^Sc^ deposits in the hippocampus and entorhinal cortex		
	4) Severe diffuse PrP^Sc^ deposits in the cerebellar molecular and granular layers, occasional small PrP^Sc^ aggregates in the cerebellar granular layer; Marked global atrophy of the cerebellum		
	5) Linear and curvilinear PrP^Sc^ deposits in the midbrain and cerebellar dentate nucleus		

* There were six E200K-129 V NPDPSC cases (A-F). No clinical information was available for Cases A and B. Case D, an 85 yo woman with dementia and known family history of E200K presenting with confusion but no other known clinical history, was not included in the table due to limited clinical information. Her MRI reportedly showed atrophy, chronic, microvascular ischemic change. Pathology revealed senile plaques of Alzheimer’s disease and mild vacuolation throughout the neocortex. Vacuolation was mild in the hippocampus, moderate in the entorhinal cortex and putamen. The cerebellum showed vacuolation without atrophy and also had focal PrP^Sc^ deposit with a perpendicular orientation to the cerebellar pial surface. Immunostaining was similar to that of Patient C but no plaque-like PrP formations were observed overall. PrP^Sc^ was type 2; N/A = not available.

**Table 4:** Summary of histopathological and molecular comparison between the E200G and E200K cases

	**E200G-129V (M)**				**E200K-129V(M)**[17]				**E200K-129M(V)**[1,8,14]			
**Pathology**^**a**^**\ Brain regions**	**A**	**B**	**C**	**D**	**A**	**B**	**C**	**D**	**A**	**B**	**C**	**D**
Cortex	+++	+++	+++	++ /+++	++/+++^c^	+++^b^	n/a	+++^b^	+++^d^	+/++^e^	+++^d^	+++^d^
									+/++^e^		+/++^e^	+/++^e^
Striatum	+++	+++	+++	++ /+++	++/+++^c^	+++^b^	n/a	+++^b^	++^d^	++^e^	++^d, e^	++^d, e^
									++/+++^e^			
Hippocampi	++/+++	-	-	-	+^b^	++/+++^b^	n/a	+++^b^	++^e^	+^e^	+^e^	+^e^
										++/+++^b^		
Thalamus	+++	+/++	+++	+++	++/+++^c^	+^b^	-^b^	-^b^	++/+++^e^	++^e^	++^e^	++^e^
										+^b^	-^b^	-^b^
Cerebellum	+	+	+/++	++	++/+++^b^	+++^b^	n/a	+++^b^	++^d^	+^e^	++^d^	++^d^
					+++^c^				++^e^		+^e^	+^e^
Vacuole size, mean		14. 9				11.9				11.8		
(range μm)		(4–108)^b^				(4–72)^b^				(4–69)^b^		
PrP^Sc^ staining pattern	Finely granular synaptic +++				Finely granular synaptic ++/+++^b, c^				Finely granular synaptic +++^e, f^			
	Plaque-like +				Plaque ++/+++^b, c^				Plaque-like ++/+++^e^, +^f^			
	Intra-neuronal +				Intra-neuronal + ^b^				Intra-neuronal ++/+++^e^			
Regions with linear and curvilinear arrays of PrP^Sc^	Striatum, hippocampi				Put, GP(20%)				Put, GP			
					SN, PAG				(12.5%)			
	SN				(67%)				CA4 of hippo (37.5%)			
	MT				CDN (100%)^b^							
									SN, PAG			
	PAG								(100%)			
	MION											
	CDN											
									CDN (80%)^b^			
PrP^Sc^ type	2				2^c^				1 (47%),			
					2(86%), 1 + 2(14%)^b^				2 (26.5%),			
									1 + 2 (26.5%)^b^			

*Abbreviations and Definitions:*^a^*A* PrP^Sc^ deposit, *B* Vacuolation, *C* Neuronal Loss, *D* Reactive Astrocytosis, *SN* Substantia nigra, *MT* Midbrain tegmentum, *PAG* Periaqueductal gray matter, *MION* Medullary inferior olivary nucleus, *CDN* Cerebellar dentate nucleus, *Put* Putamen, *GP* Globus pallidus, - absent, + mild, ++ moderate, +++ severe, ^b^*NPDPSC* analysis in this study, ^c^ from reference [17] (n = 1), ^d^ from reference [1], ^e^ from reference [14] (n = 14), ^f^ from reference [8], *n/a* not available.

Notably, all three NPDPSC E200K-129V(M) cases showed marked atrophy of the cerebellum (global in two cases and focal in one case), which exhibited severe diffuse, fine synaptic PrP^Sc^ deposits (occasional small PrP^Sc^ aggregates present but no plaque-like formation), vacuolation and reactive astrocytosis in the cerebellar cortex. Two NPDPSC E200K-129V(M) cases which did not have clinical information and were used for histopathological exam, also showed very similar cerebellar pathology to these three cases (not shown). One case which was not included in this clinical and pathological comparison due to insufficient clinical information available (Table 3, Legend) showed focal PrP^Sc^ deposits with a perpendicular orientation to the cerebellar pial surface. Table 4 compares pathological findings of our E200G case and E200K-129V(M) and E200K-129M(V) (E200K; taken from the literature and the NPDPSC cases).

For a comparative molecular study of PrP^Sc^, we included NPDPSC brains from E200K-129V(M), E200K-129M(V), sCJDMM1 and sCJDVV2. E200K-129V(M) PrP^Sc^ co-migrated with the PrP^Sc^ type 2 of E200G-129V(M). A fragment of ~17 kDa was detected in the cerebellum of two E200K-129V(M) (one case shown in Figure 6a, line 3), but not in our patient, nor in E200K-129M(V). PrP^Sc^ from one E200K-129M(V) case, however, migrated about 1 kDa slower, to ~20 kDa (Figure 6a, lines 9–11) matching PrP^Sc^ type 1, whereas another E200K-129M(V) case showed type 2 PrP^Sc^ (Figure 6a, lines 12–13). To better understand the spectrum of PrP^Sc^ types in E200K CJD, we selected additional NPDPSC E200K cases for whom Western blots were available. Among seven E200K-129V(M) cases, 86% were type 2 (n = 6) and 14% were both types 1 and 2 (n = 1) and all E200K-129V(V) cases were type 2 (n = 4). Among the E200K-129M(V) cases, 47% were type 1 (n = 9), 26.5% each were type 2 (n = 5) and both types 1 and 2 (n = 5). Among the E200K-129M(M) cases, 91% were type 1 (n = 49) but 9% were both type 1 and 2 (n = 5) (Table 5).

**Table 5:** **Comparison of disease duration and age at disease onset, and PrP**^**Sc **^**types between different E200K haplotypes**

**Haplotypes (129 cis)**	**Trans codon**	**n**	**Type 1 PrP**^**Sc**^		**Type 2 PrP**^**Sc**^		**Type 1 & 2 PrP**^**Sc**^		**All PrP**^**Sc **^**Types**	
			**%**	**Disease duration**	**%**	**Disease duration**	**% types**	**Disease duration**	**Disease duration**	**Age at onset**
	**129**		**type 1**	**(mean ± SD; range)**	**type 2**	**(months, mean ± SD; range)**	**1 and 2**	**(months, mean ± SD; range)**	**(months, mean ± SD; range)**	**(years mean ± SD; range)**
**E200K-129V**										
	M	7	0	-	86%	8 ± 1	14%	10	8 ± 1	56 ± 12
					(n = 6)	(7–9)	(n = 1)		(7–10)	(37–67)
	V	4	0	-	100%	10 ± 6	0	-	10 ± 6	54 ± 12
					(n = 4)	(2–17)			(2–17)	(37–66)
All		11	0	-	91%	9 ± 4	8%	10	**9 ± 4 **^**a**^	55 ± 11
					(10)	(2–17)	(n = 1)		**(2–17)**	(37–67)
**E200K-129M**										
	M	54	91%	4 ± 2	0	-	9%	4 ± 2	**4 ± 2 **^**b**^	59 ± 11
			(n = 49)	(1–12)			(n = 5)	(2–7)	**(1–12)**	(40–85)
	V	19	47%	**5.5 ± 3 **^**c**^	26.5%	**16.5 ± 8 **^**c**^	26.5%	7 ± 4	**9 ± 7 **^**b**^	63 ± 7
			(n = 9)	**(3–10)**	(n = 5)	**(6.5-29)**	(n = 5)	(2–11)	**(2–29)**	(53–76)
All		73	79%	4 ± 2	7%	16.5 ± 8	14%	5 ± 3	**5 ± 4.5 **^**a**^	60 ± 10
			(n = 58)	(1–12)	(n = 5)	(6.5-29)	(n = 10)	(2–11)	**(1–29)**	(40–85)

*Abbreviations and Definitions:n* Number of cases, Bold denotes subgroups with significant differences. ^a^ E200K-129V had longer duration than E200K-129M (p = 0.029); ^b^ E200K-129M(V) lived longer than E200K-129MM (p = 0.004); ^c^ Among E200K-129M(V), PrP^Sc^ type 2 lived significantly longer than type 1 (p = 0.036). All other groups did not differ significantly from each other in disease duration or age at onset.

Having found that each group had diverse PrP^Sc^ types, we examined whether codon 129 polymorphism and PrP^Sc^ types affected clinical manifestations (age at disease onset and disease duration) of E200K CJD (Table 5). We found that the E200K-129V cohort had the longer disease duration than the E200K-129M (9 ± 4 *vs*. 5 ± 4.5 months, p = 0.024). But among the E200K-129V cohort, there was no difference in the duration between 129V(M) and 129V(V) cases (8 ± 1 *vs.*10 ± 6, p = 0.75). Interestingly, among the E200K-129M cohort, cases with 129M(V) had the longer duration than those with 129M(M) (9 ± 7 *vs.* 4 ± 2 months, p = 0.004). We also found that among the E200K-129M(V), cases with PrP^Sc^ type 2 had the longer duration than those with type 1 (16.5 ± 8 *vs.* 5.5 ± 3 months, p = 0.036). But the age at disease onset was not affected by either codon 129 polymorphism or PrP^Sc^ types in E200K patients of both haplotypes (E200K-129V *vs.* E200K-129M, p = 0.21; E200K-129V(M) *vs.* E200K-129V(V), p = 0.83; E200K-129M(V) *vs.* E200K-129M(M), p = 0.18; E200K-129M(V) PrP^Sc^ type 2 *vs.* E200K-129M(V) PrP^Sc^ type 1, p = 0.45).

On the other hand, the representations of the di-, mono- and unglycosylated isoforms of PrP^Sc^ in E200K-129V(M) and -129M(V) were similar to that of our E200G patient, characterized by the prominent representation of diglycosylated PrP^Sc^ and underrepresentation of unglycosylated PrP^Sc^ species (Figure 6a-b); the amounts of the three glycoforms (di-, mono-, and unglycosylated PrP^Sc^), expressed as percent distribution of each form, were 51:38:11 in our patient, 52:39:9 in E200K-129V, 49:40:11 in E200K-129M-PrP^Sc^ type 1, and 54:38:8 in and E200K-129M-PrP^Sc^ type 2 (Figure 6b). As expected, the PrP^Sc^ glycoform ratios associated with our patient and the E200K differed from those of sCJDMM1 and sCJDVV2 in which the monoglycosylated PrP^Sc^ was predominant (sCJDMM1: 21:46:33; sCJDVV2: 35:49:16) (Figure 6b).

### Materials and methods

The work in this paper was approved by the UCSF Committee on Human Research and University Hospitals Case Medical Center Institutional Review Board. The proband and her sibling were subjects in a UCSF research study on human prion disease that includes an extensive standardized clinical evaluation. Autopsy and sampling of material for histopathological analyses of various brain regions were performed as previously described [18] including the thalamus and cerebellum. One half of the brain was frozen, the other fixed in formalin [19]. Immunohistochemistry on fixed sections and histoblot on frozen sections were performed as previously described [20-24]. Histologic sections were evaluated and assessed for PrP^Sc^ deposition with 3F4 antibody (from Dr. Stanley Prusiner), vacuolation with hematoxylin and eosin staining, reactive astrocytosis with GFAP staining (polyclonal rabbit, catalog # Z0334, Dako, Carpinteria, CA), and dendritic staining with MAP-2 antibody (polyclonal rabbit, catalog # AB5622, Millipore, Billerica, MA), as previously described [18,19,22]. Neuronal loss was judged by visual assessment as absent, mild, moderate or severe. The presence of other proteinopathies was assessed by antibodies for hyperphosphorylated tau (CP13, from Peter Davies), β-amyloid (mouse anti-β-amyloid monoclonal Ab (mAb), 4G8, catalog# NE1002, Millipore), α-synuclein (mouse mAb, catalog# ab27766, Abcam, Cambridge, MA), TDP43 (rabbit polyclonal, catalog # 10782-2-AP, Proteintech Group, Inc. Chicago, IL), and ubiquitin (rabbit polyclonal, catalog # Z0458, Dako), as previously described [25].

*PRNP* analyses and Western blot for typing of the proteinase K (PK)-resistant PrP^Sc^ were performed by the National Prion Disease Pathology Surveillance Center (NPDPSC) (Cleveland, OH) [2]. For a comparative molecular study of prion typing, frozen brains were collected by the NPDPSC from genetic prion disease (gPrD) E200K-129 V (n = 4), E200K-129M (n = 3), sCJDMM1 (n = 1) and sCJDVV2 (n = 1). All E200K cases selected from the NPDPSC database were heterozygous at codon 129; Due to the rarity of E200K-129 V cases, all available cases were used without applying any inclusion/exclusion criteria. E200K-129M had age of disease onsets similar to that of our E200G case, with disease durations felt to be representative of most of the E200K NPDPSC cohort. For histopathological comparison, immunohistochemistry (IHC) also was performed on E200K-129V (n = 6) and E200K-129M (n = 9) that had disease durations with a range of 7 to 10 and 3 to 29 months, respectively. All E200K-129V cases were analyzed for IHC. The nine E200K-129M(V) (trans V) cases were chosen for IHC to be representative of the E200K-129M population. The entire NPDPSC E200K-129M(V) cohort has a mean disease duration of 9 ± 7 (SD) months. One long duration E200K-129M(V) case, with a disease duration of 29 months, was included because it was close in duration to our E200G case; thus the mean disease duration of these nine E200K-129M(V) cases was 12 ± 7 (SD) months (10 ± 4 [SD] months without the long-duration 29 month case).

#### Preparation of the brain homogenates and Western blot analysis

Brain homogenates (BH) (20% w/v) were prepared in 1X Dulbecco’s phosphate buffer saline (DPBS), pH 7.4, then mixed with an equal volume of 2X lysis buffer containing 100 mM Tris (100 mM NaCl, 0.5% Nonidet P-40, 0.5% sodium deoxycholate, 10 mM EDTA, 100 mM Tris–HCl, pH 8.0). The BH was centrifuged at 1000 g for 5 minutes and the collected supernatant (S1) was digested with 10 U/ml proteinase K (PK) (1 U/ml equal to 20 μg/ml PK) at 37°C for 1 hour. Samples were loaded onto a 15% Tris–HCl polyacrylamide precast gel, transferred, then incubated with the primary prion protein antibody 3 F4 (1:40,000) for 2 hours. The prion protein was visualized by the Odyssey infrared imaging system. Densitometric analysis was performed with Odyssey application software V3.0 (LI-COR Biosciences).

#### Vacuole size determination and statistical analysis

The diameter (μm) of the vacuoles from the cerebral cortex of E200G, E200K-129M (n = 3), and E200K-129V (n = 3) was measured by the software Image-Pro Plus (Media Cybernetics, Inc.). Statistical analysis was performed with GraphPad Prism 6.0 using the nonparametric Mann Whitney test with Bonferroni correction of the level of significance value (α) which is equal to 0.016. For comparisons of disease duration and age of onset of various E200K haplotypes, Student t test with Welch's correction was used.

## Conclusions

To our knowledge this is the first report of an E200G mutation associated with a CJD phenotype. Several reasons strongly suggest that this mutation is pathogenic. Firstly, it is in the same codon as the most common *PRNP* mutation, E200K causing genetic CJD. Secondly, the change from a glutamate to glycine is non-conservative, substituting a large, acidic amino acid with a side-chain, with a much smaller, non-acidic amino acid without a side-chain that might affect the propensity for misfolding of PrP. Thirdly, some of the uncommon pathological features and PrP^Sc^ glycosylation pattern argue against this case being sCJD; prominent involvement of the deeper cortical layers observed in our patient is rare in most sCJD, except sCJD VV2. Although our patient was MV2, there were no amyloid kuru-type plaques, which is in contrast to sCJD MV2 in which amyloid kuru-type plaques are typically present [22]. Both of these findings (involvement of the deeper cortical layers and and the absence of amyloid kuru-type plaques), however, are frequently observed in E200K genetic prion disease (gPrD) [26]. Our patient had type 2 PrP^Sc^ but with the underrepresentation of the unglycosylated form (Figure 4), another characteristic of genetic prion disease, including CJD E200K and FFI, but not sCJD [27-29]. Lastly, although the father was reported to have an “alcoholic dementia” with a very rapid decline, we suspect that he died of gPrD. Not uncommonly in our gPrD families, we find that prior to a *PRNP* mutation being identified, some family members with gait ataxia and dementia were assumed to have had alcoholism causing cognitive impairment and gait problems.

To compare clinical and pathological phenotypes of our patient with those of E200K, we conducted extensive literature review as well as new pathological and molecular analyses on the E200K cases selected from the NPDPSC database. One might expect for E200G-129V, type 2 PrP^Sc^ to have similarities to the very rare E200K-129V, type 2 PrP^Sc^. We are aware of only two previously published cases of the E200K-129V; one V(M), like our patient, and the other V(V); both also had type 2 PrP^Sc^ that also were primarily diglycosylated [17,29]. The codon 129V(V) E200K patient was a 66-year-old woman with a 15.5 month course, beginning with one year of vertigo and ending after 3.5 months of rapidly progressive dementia. Similar to our patient she had no myoclonus; her EEG, however, showed focal triphasic spikes. MRI reportedly only showed ventricular enlargement, and probably did not include FLAIR or DWI [29]. The E200K-129V(M) case was a 67-year-old woman with a six-month course, beginning with gait ataxia and then rapidly progressive dementia at 4 months, but died of a pulmonary embolism. She had focal myoclonus in her later stages, but no PSWCs on EEG and MRI findings were not reported [17]. Our patient’s age of onset was a decade younger (57 years) and had a longer duration (30 months), than the E200K-129V cases, but similarly had early gait disturbance and late dementia [17,29]. Despite our patient’s most prominent, early and debilitating symptom of ataxia, her cerebellum was very mildly affected pathologically (Table 2). In contrast, PrP^Sc^ accumulation was noted in the cerebellum of both published E200K-129V cases; the E200K-129V(V) patient with predominant plaque-like PrP deposits [29] and the E200K-129V(M) patient with focal PrP^Sc^ deposits with a perpendicular orientation to the pial surface in the cerebellar molecular layer [17].

Compared with the NPDPSC E200K-129V(M) cases (mean age at onset 56 ± 12 years [range 37–67 years], mean duration 8 ± 1 months [range 7–9 months], Table 5), however, our E200G patient had a similar age at onset, but a much longer disease duration. Common features between the E200G case and three well-characterized cases of NPDPSC E200K-129V(M) (Table 3) are initial gait difficulty followed by memory decline, striatal involvement on brain MRI, slowing on EEG, elevated CSF tau level, and type 2 PrP^Sc^. But, cortical ribboning which was mild in our E200G patient, was not observed in the NPDPSC E200K-129V(M) and myoclonus was reported in one NPDPSC E200K-129V(M) case (Table 3). In our experience, however, in most E200K cases which are reported to not have cortical ribboning on DWI/ADC MRI, we identify cortical ribboning. A majority of MRIs in CJD, unfortunately, are misread [16,30]. Pathologically, cerebellar involvement was much more severe in the NPDPSC E200K-129V(M) cases than our case. Only one of the six NPDPSC E200K-129V(M) cases examined histopathologically (Table 3, Legend) showed the similar focal PrP^Sc^ deposits perpendicular to the cerebellar pial surface to the published E200K-V(M) patient [17]. The other five NPDPSC E200K-129V(M) cases (Table 3 and not shown) did not show either plaque-like PrP^Sc^ deposits or focal PrP^Sc^ deposits with a perpendicular orientation in the cerebellar cortex. We found marked atrophy in their cerebella, however, accompanied by severe diffuse PrP^Sc^ deposits and severe vacuolation and reactive astrocytosis, which are in great contrast to the mild cerebellar involvement of our E200G patient (Tables 2 and 3). In addition, we detected another fragment of ~17 kDa of PrP^Sc^ in the cerebellum of the two NPDPSC E200K-129V(M) cases with Western blotting, which was not observed in our patient (Figure 6). Whether the cerebellar presence of the 17 kDa fragment is a distinctive feature of E200K-129V(M) remains to be determined. There are clearly overlapping as well as distinguishing features between our case and the E200K-129V(M) gPrD patients.

Although our patient was cis 129V, she had many features in common with the most prevalent form of E200K, with 129M cis (E200K-129M). The age at onset (57 years) in our case is consistent with those of E200K-129M, with a published mean age at onset 58 years (range 33–84 years) [5,8,10] and those of NPDPSC with 60 ± 10 years (range 40–85 years) (Table 5). Our patient’s duration of illness (30 months), however, is longer than the published mean duration of E200K (6; range 2–41 months) and those of NPDPSC (5 ± 4.5; range 1–29 months) (Table 5) but within the published range [5,8,10]. Brain MRI findings of the predominant striatal involvement and mild cortical ribboning observed in our patient (Figure 2) are also common in E200K-129M [31,32]. Pathologically, the pattern of PrP^Sc^ deposits was diverse in our patient (synaptic, coarse granular, and plaque-like deposits) overlapping with those in Slovakian E200K-129M (synaptic pattern mainly in 129MM and granular or plaque-like deposits in 129MV) cases [8]. Involvement of deep cortical layer and no amyloid kuru-type plaques of PrP^Sc^ were both present in our patient and also are in E200K-129M (Figure 3) [26].

Our case also had several differences from those typically found in E200K-129M. Our patient initially presented with gait ataxia followed by dementia, whereas dementia typically occurs first in E200K-129M [5,8,10]. Our patient did not develop myoclous or EEG PSWCs, which are very common in published E200K-129M cases (myoclonus in 73% and PSWCs in 75%) [33,34]. A characteristic stripe-like pattern with a perpendicular orientation of PrP^Sc^ deposits in the cerebellum noted in published E200K-129M [14,26,35] cases was not observed in our case. In addition, our patient had type 2 PrP^Sc^, whereas E200K-129M cases have type 1, 2 or mixture of both types [14,29,36]. Other differences between our patient and E200K cases examined were the larger vacuole sizes (the mean diameter of the vacuoles from E200G significantly larger than those of the NPDPSC E200K-129M and E200K-129V), no vacuolation in the hippocampus (despite abundant PrP^Sc^ staining), and the severe involvement of the thalamus (severe atrophy, PrP^Sc^ deposits, neuronal loss, and reactive astrocytosis, and mild vacuolation) (Figure 3). Vacuolation in the hippocampal pyramidal cell layer was observed in the NPDPSC E200K-129M (6 cases out of 7 cases examined) and -129V cases (all 6 cases examined). The thalamic changes noted in our case have been reported only in one case of E200K-129M [37] and commonly seen in sporadic and familial fatal insomnia [38,39].

In one previous study, Hτ positive neuritis (93.3%), parenchymal Aβ (53.8% but only occasional neuritic plaques) and Lewy type α-synuclein pathology (15.4%) were reported in E200K (all codon 129 polymorphisms, except no V(M) were represented) [14]. We found Hτ-positive neuronal cell bodies and neuropil threads in the entorhinal cortex of our patient (Figure 5). This distribution of Hτ was found to be characteristic of great majority of sCJD and familial CJD (fCJD) cases analyzed at our center (DeArmond, Tousseyn, Bajsarowicz, et al., in preparation). It has been reported that among non-demented subjects 20–50 years of age, 11% had limited number of Hτ’s in the entorhinal cortex and trans entorhinal cortex [40]. No Aβ plaques were found in any regions of our patient, however (Figure 5). This is consistent with our unpublished findings that only 2 of 14 cases of fCJD had AD neuropathology (DeArmond et al., in preparation). It is also consistent with the age of death of our patient (59 years), as CJD-AD cases are found to occur almost 10 years later (DeArmond et al., in preparation).

In the NPDPSC E200K cohort, cases with a valine at codon 129 and PrP^Sc^ type 2 had longer disease duration (Table 5). Considering the longer duration of our patient with E200G-129V(M), PrP^Sc^ type 2 than that of E200K-129V(M), PrP^Sc^ type 2 cases, we suspected that this novel mutation might contribute to the prolonged disease course.

One of the most unusual findings in our case was the abundant curvilinear arrays of PrP^Sc^ in various brain regions (Figures 3 and 4). To our knowledge there is only one reported CJD case (unclear if genetic or sporadic) with a similar pattern of PrP^Sc^ deposits [41]. We also observed this staining, however, in all NPDPSC E200K-129V(M) and most E200K-129M(V) cases examined histopathologically in this study (Table 4).

The subcellular localization of the PrP^Sc^ deposits to synapses and presynaptic axonal terminals might be related to transsynaptic spread of PrP^Sc^. It remains unknown whether PrP^Sc^ spreads along defined neuroanatomical pathways in human prion disease, which is the case in scrapie-injected mouse [42,43] and hamster models [44,45]. Notably, the subcortical gray matter and brain stem enriched in the curvilinear arrays of the synaptic PrP^Sc^ in our patient are known to be preferentially vulnerable regions in progressive supranuclear palsy (PSP) [46]. This is interesting as it is now realized that pathological tau aggregates might propagate by a prion-like mechanism [47-49]. A recent functional MRI (fMRI) study in PSP showed the connectivity disruption of the same dorsal midbrain tegmentum-anchored intrinsic connectivity network (ICN) (brain stem, cerebellum, striatum and cortex) [46] as was affected pathologically in our case. The overlap of the affected brain regions and some PSP-like clinical features (gait difficulty, saccadic pursuit, slowed velocity of horizontal saccades, and parkinsonism) in our patient suggested the possibility of PrP^Sc^ spreads through this PSP-related ICN.

As this was a single case, the spectrum of clinicopathological presentation of this novel mutation has yet to be determined. This case supports, however, the loss of glutamate at codon 200, and not its replacement with lysine, as the cause of PrP misfolding. As has been done with E200K [50], a mouse model of E200G might help further show the pathogenicity of this novel mutation. The penetrance of this E200G mutation has yet to be determined. We suspect that conformational changes of PrP^Sc^ caused by the E200G mutation and the rare codon 129 cis valine polymorphism might contribute to the distinct clinical and pathological findings of our patient in contrast to the E200K-129M and E200K-129V subjects.

## Consent

Written informed consent was obtained from the patient’s next of kin for the publication of this report and all accompanying images.

## Competing interests

The authors declare that they have no competing interests. Dr. Geschwind has served as a consultant for MedaCorp, The Council of Advisors, Guidepoint Global, and Neurophage.

## Authors’ contributions

MOK analyzed clinical data, designed the studies of immunohistochemistry (IHC), created figures, and drafted the manuscript. IC analyzed E200K clinical data, performed all Western blots and IHC, created figures, and edited the manuscript. AO performed IHC. JCF, KW, TS, and JSK were involved in the collection of clinical data on the E200G case. PG contributed to the collection of E200K clinical data and editing the manuscript. BMB provided interpretation of neuropsychological data and edited the manuscript. SJD performed neuropathological analysis for the E200G case. MDG supervised the study, collected and analyzed E200G clinical data and co-wrote and edited the manuscript. All authors read and approved the final manuscript.
